# Just a leader? Leadership work challenges and identity contradiction experienced by Finnish physician leaders

**DOI:** 10.1108/JHOM-10-2020-0421

**Published:** 2021-06-18

**Authors:** Sari Huikko-Tarvainen, Pasi Sajasalo, Tommi Auvinen

**Affiliations:** School of Business and Economics, University of Jyväskylä , Jyväskylä, Finland

**Keywords:** Healthcare, Physician, Physician leader, Physician leadership, Identity

## Abstract

**Purpose:**

This study seeks to improve the understanding of physician leaders' leadership work challenges.

**Design/methodology/approach:**

The subjects of the empirical study were physician leaders ( *n* = 23) in the largest central hospital in Finland.

**Findings:**

A total of five largely identity-related, partially paradoxical dilemmas appeared regarding why working as “just a leader” is challenging for physician leaders. First, the dilemma of identity ambiguity between being a physician and a leader. Second, the dilemma of balancing the expected commitment to clinical patient work by various stakeholders and that of physician leadership work. Third, the dilemma of being able to compensate for leadership skill shortcomings by excelling in clinical skills, encouraging physician leaders to commit to patient work. Fourth, the dilemma of “medic discourse”, that is, downplaying leadership work as “non-patient work”, making it inferior to patient work. Fifth, the dilemma of a perceived ethical obligation to commit to patient work even if the physician leadership work would be a full-time job. The first two issues support the findings of earlier research, while the remaining three emerging from the authors’ analysis are novel.

**Practical implications:**

The authors list some of the practical implications that follow from this study and which could help solve some of the challenges.

**Originality/value:**

This study explores physician leaders' leadership work challenges using authentic physician leader data in a context where no prior empirical research has been carried out.

## Introduction

Healthcare sector resources have been globally pushed to near breaking point by the Coronavirus disease 2019 (COVID-19) pandemic. In such crises, good management and leadership throughout the healthcare system are of crucial importance to facilitate the best use of available resources, not only to save lives but also achieve the ultimate goal of bettering the health of citizens ( WHO, 2000 , p. 23). According to previous research, engaging physicians in the leadership of health systems improves patient care and organizational performance ( Falcone and Santiani, 2008 ; Goodall, 2011 ; Perry *et al.* , 2017 ; Spurgeon *et al.* , 2015 ; Tasi *et al.* , 2017 ), affects staff well-being ( Päätalo and Kauppi, 2016 ), and above all, patient outcomes ( Onyura *et al.* , 2019 ).

If effective physician leadership provides such benefits in the medical profession and health systems, why is effective physician leadership, apparently, so hard to accomplish? After all, medical leadership has been noted to be an intrinsic component of all physicians' daily clinical work at all hierarchical levels ( Baker and Dennis, 2011 ; Edmonstone, 2009 ; Noordegraaf *et al.* , 2015 ). Regardless, even physician leaders do not primarily identify themselves as leaders/managers ( Quinn and Perelli, 2016 ; Llewellyn, 2001 ; Ong, 1998 ; Spehar *et al.* , 2015 ), but as physicians instead ( Baker and Denis, 2011 ; Llewellyn, 2001 ; Witman *et al.* , 2011 ). As Andersson (2015) argues, the leader identity is almost an antithesis to the physician identity. The challenge of physician leadership appears, therefore, as not only an issue of structure and/or competence, and those already make healthcare an especially challenging context for leaders ( Angood and Shannon, 2014 ; Leatt and Porter, 2003 ), but just as much—or possibly even more so—an identity and value challenge (e.g. Llewellyn, 2001 ).

For the reasons outlined above, building further understanding of the challenges faced by physician leaders in their leadership work is timely and relevant. In this paper, we build on the challenges experienced by a group of Finnish physician leaders trying to balance the contradictory forces of the expectation of being medical professionals and physician leaders, stemming from both systemic grounds and various stakeholders. We will provide some answers to the question of why it is so hard for physician leaders to work as “just a leader”. Our research question is as follows: which kinds of challenges emerge in physician leaders' leadership work and why? Our aim is to provide resources for developing physician leadership theory and suggest ways of resolving some of the challenges physician leaders face trying to balance the paradoxical demands of their work.

## Theoretical framework

To allow an informed analysis of the challenges met by physician leaders in their leadership work, we have built our theoretical framework to illustrate both the nature of physician leader work and some central contextual issues related to their working environment. These being the professional bureaucracy of healthcare organizations, the competing institutional logic of healthcare and professional identity-related issues, that, when combined, has an impact on physician leadership and its practice in healthcare organizations.

### Physician leadership

Leadership has many definitions but is most often perceived as “a social process of influence towards the attainment of a common goal … to achieve direction, alignment and commitment” ( Swanwick, 2017 , p. 35). However, none of the existing leadership theories have specifically defined physician leadership (see, e.g. Yukl, 2013 ). Based on the prior literature, physician leadership is mostly conceptualized as leadership work that simply combines medical and managerial work (see, e.g. Berghout *et al.* , 2017 ; Buchanan *et al.* , 1997 ; Witman *et al.* , 2011 ; Andersson, 2015 ; Quinn and Perelli, 2016 ; Llewellyn, 2001 ; Ong, 1998 ; Spehar *et al.* , 2015 ; Baker and Denis, 2011 ; Berg and Byrkjeflot, 2014 ; Byrkjeflot and Jespersen, 2014 ). However, as Quinn and Cola (2020 , p. 1492) highlight, “that physicians may not value leadership may be the most important factor in differentiating physician leadership from mainstream leadership theory”.

### Healthcare organizations as professional bureaucracies

Mintzberg (1983b , pp. 189–213; 1998 , pp. 348–349), among others, has used hospitals as an example of professional bureaucracy, a stability-oriented organization in which highly qualified professionals—physicians—have considerable control over their work. The power to influence the organization's performance and the ability to change the behaviour of others in a professional hierarchy builds on professional expertise ( Mintzberg, 1983b , p. 198). Mintzberg further argues that having power related to one's own work means being rather independent from colleagues ( Mintzberg, 1983b , p. 190). Mintzberg emphasizes that in situations where a professional must choose between profession-bound and administrative work, power is transferred to professionals focusing on administrative work. However, even then, the administrators retain their power only as long as the professionals feel the administrators are effectively serving the interests of their profession ( Mintzberg, 1983b , p. 200).

### Competing institutional logics

A recent systematic literature review of medical leadership ( Berghout *et al.* , 2017 ) concluded physician leaders' work comprises both general management/leadership activities and medicine, requiring balance between these two competing professional fields. Competing logic leads physician leaders to feel pressured into either–or decisions concerning important aspects of their work: quality of care versus efficiency ( Buchanan *et al.* , 1997 ), work autonomy versus subordination ( Andersson, 2015 ) or engaging in clinical versus managerial work ( Witman *et al.* , 2011 ). Andersson (2015) further points out that physicians at any hierarchical level are more strongly associated with their physician professional culture rather than any other organizational sub-culture.

### Professional identities in healthcare

A professional identity, the combination of attitudes, beliefs, motives, values and experiences through which people define themselves in a professional role ( Schein, 1978 ) forms over time through various interactions, such as individual experiences, formal education, feedback and co-worker behaviour. The main influences on professional identity formation are language, communication, privileges, responsibilities, community, context and setting ( Chan *et al.* , 2018 ).

As physician leaders experience meaning, satisfaction and legitimacy through their clinical work ( Quinn and Perelli, 2016 ; Llewellyn, 2001 ; Ong, 1998 ; Spehar *et al.* , 2015 ), they gravitate towards working mainly with medical issues ( Baker and Denis, 2011 ; Berg and Byrkjeflot, 2014 ; Byrkjeflot and Jespersen, 2014 ; Llewellyn, 2001 ; Witman *et al.* , 2011 ). Furthermore, according to Spehar *et al.* (2015) , physician leaders feel clinical provide the needed credibility among colleagues and the ability to influence colleagues without drawing on professional power. Consequently, it appears that medical credibility among peers is key for an effective physician leader, resulting in less time spent on leadership work for fear of losing credibility ( Llewellyn, 2001 ).

To contrast identity/role construction within healthcare, Spehar *et al.* (2015) found nurses progress into managerial roles faster (see also Bondas, 2006 ) and also feel more positive about the transition, are more fully engaged in the managerial aspects of their work and place less importance on demonstrating clinical competence. Berg (1996) argues that while physicians want to perform leadership and management work, they do not want to see it as a separate managerial discipline, but rather an extension of their own medical role ( Spehar *et al.* , 2015 , p. 361 citing Berg, 1996 ). Similarly, Summerfield (2014 , p. 252) finds that “leadership is a professional obligation of all pharmacists and not the exclusive responsibility of pharmacists who hold informal leadership roles or titles”. Physicians not considering managerial work as a career step ( Kippist and Fizgerald, 2009 ) make these contrasting findings an interesting anomaly to explore further.

An extensive study of physicians' work ( *n* = 1,233) noted that professional identity evolves constantly over a physician's career and that leader identity becomes stronger with the number of years spent in the position ( Ministry of Social Affairs and Health, 2020 ). Forouzadeh *et al.* (2018) point to education as an essential part of the formation of a professional identity. Moreover, according to Quinn and Perelli (2016) , role internalization, relational recognition and organizational endorsement are pivotal precursory elements for physician leaders' self-identity construction, which is an essential factor affecting their performance. Furthermore, Quinn and Perelli (2016) argue that successfully functioning as a physician leader may necessitate accepting a dual identity.

## Research context

As contextual effects may influence the transferability of findings of leadership practices and entrenched paradoxical tensions in health system organizations ( Onyura *et al.* , 2019 ), we provide a brief overview of the Finnish public healthcare system and its organization to offer additional context for our results.

### Finnish public healthcare

All 5.5 million inhabitants of Finland ( Statistics Finland, 2019 ) are entitled to adequate health services regardless of ability to pay or place of residence by the Finnish constitution ( Finlex, 1999 ). There are some 21,000 working-age physicians in Finland, of whom 4,026 are considered physician leaders ( Finnish Medical Association, 2016 ). Roughly half of the physician leaders participate in clinical work ( Huikko-Tarvainen *et al.* , 2019 ; Lehto *et al.* , 2003 ) and 63% of medical directors, chief physicians and deputy chief physicians of hospitals have on-call duties ( Finnish Medical Association, 2016 ). Finnish physicians have routinely advanced to formal leadership positions through career progression, often with little to no leadership training ( Huikko-Tarvainen *et al.* , 2019 ; Lehto *et al.* , 2003 ), which is also not uncommon elsewhere (see Angood and Shannon, 2014 ). While physician education does not prepare one for leadership duties, it provides a foundation for understanding the logic of healthcare service operations and gaining the trust of physician-subordinates ( Finnish Medical Association, 2013 ). Since 2009, compulsory first-line leadership training has been part of the medical specialist education in Finland ( Ministry of Social Affairs and Health, 2011 ).

The Finnish public healthcare system ranks as the best in the world in terms of quality and availability together with Iceland, Norway, the Netherlands, Luxembourg, Switzerland and Australia ( GBD 2016 Healthcare Access and Quality Collaborators, 2018 ). Furthermore, Finland outranks other countries in cost efficiency of healthcare, with an annual cost of $3,300 per person ( Global Burden of Disease Health Financing Collaborator Network, 2018 ). For efficiency, the work of physicians is of key importance: decisions regarding care lead to roughly 70% of the total expenditure of the healthcare system ( Alarotu, 2012 ). As physician leaders are involved in the daily operation of the healthcare system, a lot depends on them.

## Data and methods

A qualitative, constructivist interpretative approach ( Gibbs, 2007 ) was chosen as the research strategy because our research task involves furthering the understanding of the challenges faced by physician leaders in their leadership work. A semi-structured interview was chosen as the data collection method. The informants were invited to freely consider their work as physician leaders in terms of managerial and leadership roles and whether they face any challenges in their leadership work. The structure of the interview was guided by the research question, and the main interview themes were the content of the informants' work and challenges related to their physician leadership work.

The study was granted ethical approval by the Central Finland Care District. The physician leader informants of our study—chief physicians and heads of departments—were all employed by the Central Finland Central Hospital and invited to participate in the study by an internal email. The Central Finland Central Hospital serves a population base of 270,000 and employs 485 physicians, of which 50 are chief physicians and 75 are heads of departments ( KSSHP, 2019a , b ).

A total of 23 male and female physician leaders from 18 different specialist fields of medicine volunteered and were interviewed from April to June 2017 and July to August 2018. In total, 15 informants were chief physicians with extensive experience in the medical profession (28.5 years on average) and leadership positions (11.3 years on average), and eight of the informants were heads of departments and had comparable experience in their profession (24.25 years on average) but less experience in leadership positions (5.5 years on average). Chief physician ages ranged from 48 to 64 years, while heads of department were between 41 and 57 years.

The interviewees were duly informed of their participation being voluntary, and due consideration was given to matters related to data protection in accordance with the ethical principles applicable to research subjects ( Finnish Advisory Board on Research Integrity, 2012 ). The interviewees were informed of the purpose of the study and their right to withdraw their participation and deny the use of their data at any time during the study. The identities of the informants are hidden.

All interviews were digitally recorded, transcribed and coded to make the data more accessible for analysis. The duration of the individual interviews ranged from 22 to 88 min, resulting in 18 h and 32 min of speech in total. The transcribed interviews yielded a total of 398 A4-sized sheets of text (Calibri, 12 point, single spacing). Content analysis by thematization was selected as the method of analysis. After carefully reading through the interviews several times, the informants' replies were categorized according to emergent themes (expectations of commitment to patient work, undervaluation of leadership work and fear of losing professional credibility, and profession-based ethical pressure) and analysed by employing content analysis. The purpose of the content analysis was to obtain a description of the studied phenomenon, allowing us to connect our findings to a broader context and previous results on the subject, while in thematization, matters related to a specific theme were constructed from the data, as suggested by Eriksson and Kovalainen (2008 , pp. 187–189, 219). Informative excerpts have been carefully chosen from the data to illustrate the interpretations made. Expressions that could compromise anonymity have been edited in these excerpts, but this had no effect on the analysis or results due to the changed wording appearing only when presented.

## Results

To lead into our findings and discussion on the leadership work challenges experienced by physician leaders, we outline three sets of duties that are inseparably associated with the core content of physician leaders' work found in our earlier work ( Huikko-Tarvainen *et al.* , 2019 ) and alluded to by our informants in this study also. These duties, we argue, may be seen as feeding the identity incongruity of physician leaders in leadership work.

1. Medical professional duties – clinician identity: saving lives, traditional patient care
2. Leadership duties – leader identity: human resource championing, support and development of people, and care for the organizational members
3. Managerial duties – manager identity: ensuring smooth running of the “machinery”, looking after the efficiency, effectiveness and frictionless functioning of the work organization

As there is no coherent and established job description for a physician leader, the sets of duties and responsibilities of the physician leaders in our study varied considerably. However, the majority carried outpatient work as part of their regular duties as physician leaders. The work of the heads of departments in our study was more focused on clinical patient work than the chief physicians', but the emphasis in their responsibilities varied as well. Consequently, there is an inherent imbalance in the job description of physician leaders which generates challenges in all areas of their profession, especially their leadership duties ( Marchildon and Fletcher, 2016 ). Next, to answer the first part of our research question about the kinds of challenges physician leaders face in their leadership work, we will analyse them in more detail.

### Challenge I – expectations of commitment to patient work

The working environment and institutional logics of healthcare imply, favour and sometimes take as self-evident that everyone with medical education—physician leaders included—carry out patient work. Some interviewees experienced this implicit demand put on them by varying stakeholders and a pressure to prioritize patient work over leadership work, the obvious challenge being the intertwining responsibilities of a clinician and a leader and how to balance the two.

I often have to explain that, eh, what is it actually that you do, are you doing real work at all … no boss in any other line of business thinks that leading was not real work … that I can be a full-time leader without having to explain every day that I don't do patient work … Leadership as such is a value, that someone is a leader even though not being involved in patient work. (I35)

When describing their work and its distinctive features, the interviewees routinely mentioned a dichotomy between patient work and managerial/leadership work. When speaking about aspects related to managerial or leadership duties, physician leaders did not make any distinction between managing people (leadership) and managing things, but often referred to both as simply “non-patient work” instead. The non-patient work discourse covered any duties that lacked a medical aspect. This is indicative of the deep commitment to, and the general appreciation of, patient work and its innate central significance to the physician profession ( Spehar *et al.* , 2015 ). It also broaches the importance of constantly proving one's clinical competence as part of the leader role. At the same time, the dichotomy can be seen to reflect the implicit inferiority physicians see in managerial/leadership work, which constitutes both a stark identity discrepancy and a value conflict for physician leaders.

### Challenges II and III – undervaluation of leadership work and fear of losing professional credibility

Physician leaders' work fluctuates between patient work and leadership work. Our informants perceived the leadership work they performed as undervalued by their peers, in relation to patient work, and expressed hope for leadership work becoming of equal merit to patient work. Meriting leadership work as “real work” was called for, both for the work community and physicians, and yet confidence in the value of leadership work was questioned.

It would be nice if someone here appreciated leadership work as such. That its value would also be recognized among physicians—if we do not show appreciation, then how anyone else could be expected to appreciate physician leaders and their work in the organization. (I35)

That it [leadership] would be openly recognized as a demanding duty and it would be openly stated that it [leadership] is demanding. Moreover, that it would be clearly defined whether the position involves any clinical work and if so, how much, or whether it is a full-time post. That the position would, sort of, be truly recognized as a leadership position. (I27)

The physician leaders in our study perceived a lack of recognition and appreciation for their leadership work and clearly felt a need to explain and convince the other healthcare staff, including their medical colleagues and even themselves, that their leadership work is as real and important to the organization as clinical work. This is consistent with the findings of Styhre *et al.* (2016) , where leadership work was viewed as less rewarding than clinical work, and physicians therefore preferred leaders who were perceived as skilled clinicians. This puts major self-affirmation pressure on those committing to leadership work and further intensifies the struggle between leadership versus clinician identities faced by physician leaders.

Furthermore, the physician leaders considered leading other physicians demanding because those being led might have more advanced medical skills than the physician leaders themselves. This was perceived as a challenging setting best coped with by possessing good clinical skills in the speciality under one's leadership.

This is leadership of medical experts…You always have to remember that some of the subordinates are wiser in medical knowledge than their leaders. (I3)

It can be a difficult situation when you have highly experienced physicians as your subordinates. If you do not already have enough merit [in clinical skills], you are not very well equipped to do the [leadership] job then. You have no way of gaining authority, but if it is the other way around, this succeeds quite nicely, even easily. A lot is forgiven. (I37)

Based on our data, the physician leaders believed possessing good clinical skills would compensate for shortcomings in one's leadership skills. While this may work towards becoming an even better clinician, such a state is detrimental for leadership skill development. For instance, Morrow *et al.* (2014) and Giri (2017) found that physicians would benefit from leadership development in order to become more effective at their job. Leadership development would help boost not only “technical” skills in the field but also the leadership identity of physician leaders.

### Challenge IV – profession-based ethical pressure

A physician leader's professional identity and work ethic are strongly attached to the medical profession upon which their leadership also largely relies, as well as the institutional logic of healthcare. Switching over from patient work to leadership work may also be seen as abandoning medicine ( Styhre *et al.* , 2016 ), accompanied by uncertainty about the value of a physician who does not do any patient work.

A physician just needs to be on the frontline doing the work. And even though the physician leader can perhaps no longer be top-notch in his own specialty, because leadership responsibilities take so much time, he can, by his own work, set an upright example of the way we work here. (I11)

Being able to commit enough to leadership work was perceived as difficult at times because patient work seemed to take priority over everything else due to the ethical obligations associated with the profession.

If necessary, I cancel meetings or set other leadership duties aside and do them in the evening, or over the weekend, as our strategy is that the patient comes first… and if a physician falls ill, I get out there to back up, but I also want to lead from the frontline, and I want to know what's going on in the field. (I44)

Physician leaders apparently felt a sense of guilt when not carrying out patient work as laws and ethical guidelines to be complied with after assuming the leadership position are not lifted. Furthermore, as the excerpts above demonstrate, in addition to professional ethics, a personal commitment to leading by example and being informed appear as strong motivators for physician leaders to actively engage in clinical work. In terms of physician leaders' identity struggles, the ethics-driven clinician identity would appear to be subordinate to the leadership identity.

## Discussion

To better understand these challenges and their underlying reasons emerging in physician leaders' leadership work related to why working as “just a leader” is challenging, we dig deeper into five dilemmas linked to the identity struggle physician leaders are constantly experiencing in their work. This provides informed answers to the second part of our research question: why?

### Dilemma I – identity ambiguity, leader and/or physician?

Indicative of the non-routine and situational nature of leadership work ( Huikko-Tarvainen *et al.* , 2019 ; Heilmann, 2018 ), our findings suggest considerable daily and weekly variation in the work content of one and the same chief physician. This causes ambiguity and challenges in allotting time for clinical and leadership work, similar to what Spyridonidis *et al.* (2015) and Witman *et al.* (2011) have noted. This constant duality causes fluctuation not only between the two different work roles but also between the physician and leader identities, similar to Andersson's (2015) findings. Furthermore, our study corroborates previous observations (see Witman *et al.* , 2011 ; Andersson, 2015 ; Spyridonidis *et al.* , 2015 ) on the physician leader's job as being multidimensional hybrid work containing contradictory role expectations.

However, the professional identity challenge can also partly originate from other grounds than the duality of job roles. As noted by Spehar *et al.* (2015) , nurses seem to adopt leadership roles with relative ease, whereas physicians have been found to face identity challenges when working as leaders. The professional identity challenge may be related to the length of education and number of working years. As Forouzadeh *et al.* (2018) point out, professional education is essential in the formation of professional identity. Therefore, individuals may professionally identify themselves not only through their education and work but also through their own profession.

The longer the education and working period, the stronger the feeling of belonging to a profession and its professional identity is likely to become. In Finland, the length of nursing education is 3.5 years ( Degree Programme in Nursing, 2021 ), while the length of a doctor's medical education is 6 years; becoming a specialist doctor takes another 5–6 years ( Medical education program, 2021 ). All physician leaders of public hospitals in Finland are specialist doctors. Because of their extended medical education and the number of years spent working as physicians, physician leaders have had much longer to identify as physicians rather than leaders, which may lead to a more intense identity ambiguity challenge.

### “One of us” talk feasibly supporting the leader identity

Based on our findings, the basic starting point among physicians is that they strongly prefer to be led by another physician. This way, the physician leader becomes a recognized member of a shared reference group; one of “us” in the professional bureaucracy ( Mintzberg, 1983a , b , 1998 ). Being a physician is often perceived as a calling, and as such, working for the greater good, which in turn offers a sense of meaning and purpose for members of the profession. It is therefore likely that leading one's peers sets the tone for leadership behaviour, as does the fact that members of the medical profession, while varying in their level of expertise and experience, share a common goal: preserving life. This relates to the findings of Haslam *et al.* (2011) about effective leadership on a more general level; an aspiring leader should emphasize the common characteristics of themselves and those led instead of seeking to stand out from their subordinates.

Additionally, in light of previous research, medical education and training is a necessary precondition for working as a physician leader ( Styhre *et al.* , 2016 ; Ahlblad, 2014 ; Wrede *et al.* , 2016 ). When being recognized by peers as one of “us”, physician leaders can narrow the gap between medicine and management/leadership and therefore function as a bridge between the two worlds as suggested by Hoff (1999) and Llewellyn (2001) , and by doing so, remedy the division argued to plague the development of healthcare (see Witman *et al.* , 2011 ). Moreover, in order to evaluate the skills, quality of work, job performance and supplementary education needs of clinicians, medical expertise is required of their leaders ( Finnish Medical Association, 2014 ).

To summarize, the first dilemma for physician leaders to solve is clearly bound to professional identity. Physicians feel uncertain about the value of a physician who does not do any patient work, but at the same time, they want their leaders to be physicians.

### Dilemma II – once a doctor, always a doctor

Based on our findings, the culture in the Finnish healthcare organizations does not yet appear to be ready to accept physicians who relinquish their clinical work, similar to what previous studies have found in various other national contexts ( Buchanan *et al.* , 1997 ; Dickinson and Ham, 2008 ; Kippist and Fitzgerald, 2009 ; Mo, 2008 ). Healthcare organizations expect physician leaders to continue their clinical work because physician leaders are, primarily, qualified medical professionals. This culture of stability in healthcare organizations as professional bureaucracies (see Mintzberg, 1983b , pp. 189–213; Mintzberg, 1998 , pp. 348–349) does not help accepting and respecting physician leaders as “just” formal leaders, meaning these challenges are not only a professional identity matter (cf. Andersson, 2015 ) but an organizational one. As professional expertise allows power in the professional hierarchy ( Mintzberg, 1983b , p. 198) and independence in relation to colleagues ( Mintzberg, 1983b , p. 190), physician leaders may not want to lose their physician identity and the power it brings. Assuming a “just a leader” identity might lead to a loss of independence and power in the organization.

This second dilemma, related to healthcare organizations and their organizational culture, is the systemic expectation of all physicians, including physician leaders, to undertake patient work. This is because physician leaders are trained medical professionals, and first and foremost physicians with a deep commitment to a patient work. This leads to difficulty combining patient work and leadership work in the same position, further contributing to the identity discrepancy.

### Dilemma III – compensation of shortcomings in leadership skills

The study finds a deep commitment to not only the ethics of the physician profession but also to patient work. Similar to the findings of Spehar *et al.* (2015) , the physician leaders in this study feel that they gain credibility, legitimacy, authority and respect from their medical colleagues only by doing patient work. Possessing recognized clinical skills allows positional power but needs continuous peer confirmation (see also Quinn and Perelli, 2016 ; Llewellyn, 2001 ; Ong, 1998 ; Spehar *et al.* , 2015 ), leading to the noted fear of losing respect among physicians if one becomes “just” a leader. In addition, as a first new finding, our study uncovers that good clinical skills compensate for shortcomings in leadership skills. However, for leadership skill development, this kind of compensation appears detrimental.

Because mistakes in patient work can be fatal, a culture of clinical faultlessness prevails in healthcare ( Kivinen, 2008 , p. 81). Because of this, physician leaders do not want to fail in leadership work either and can compensate for shortcomings by taking part in patient work for which they are highly trained and able to excel in. However, instead of compensating for handicaps in leadership skills through clinical skills, more emphasis should be placed on leadership education and leadership work to allow physicians to benefit from leadership development ( Morrow *et al.* , 2014 ; Giri, 2017 ).

The fact that excellent clinical skills are seen to compensate for shortcomings in leadership skills only encourages physician leaders to continue, and try to excel at, patient work alongside their leadership work. This is counterproductive for the development of physician leaders in being better leaders of their subordinates or better managers trying to ensure the efficiency, effectiveness and frictionless functioning of the work organization.

### Dilemma IV – “non-patient work” talk diminishing the leader identity

A physician leader's work appears as management of a highly qualified expert's work (cf. Heilmann, 2018 ). When describing their duties, physician leaders routinely refer to leadership/managerial work as “non-patient work”, which is the second new finding of our study not mentioned in the previous literature. Furthermore, they did not make any meaningful distinction between the leadership and management aspects of their work. Management—including administrative responsibilities, legal considerations and national operating principles with their distinctive features—is more clearly a set of phenomena specific to a certain healthcare system, whereas leadership—including supporting, teaching and developing the competence of one's subordinates—is more a universal phenomenon. The term “non-patient work” reflects the strong presence of patient work in physician leaders' daily work and the doctor's profession. This leads to a situation where work tasks other than patient work—leadership and management work—are valued against patient work and appear inferior.

This dilemma, linking to the former three, is that leadership work is habitually referred to as “non-patient work” by physician leaders themselves as well as others in healthcare organizations. This represents a deeply held commitment to, and a general high regard for, patient work, which naturally has an innate central significance to the physician profession but also increases the importance of proving one's clinical competence as part of a leadership role. At the same time, this dichotomy can be seen to reflect the apparent inferiority of managerial/leadership work as part of physician leadership, which is not helpful in terms of the identity formation of physician leaders or motivating individuals to take up the challenge of becoming a physician leader and developing abilities as one.

### Dilemma V—ethical obligations to follow

The third new finding in our study is that physician leaders not only carry out exactly the same clinical work as their subordinates, but they often go beyond the call of duty by cancelling meetings or otherwise delaying leadership work if more physicians are needed to take care of patients. This points towards the binding ethical obligations of the medical profession. Physician leaders not only follow the “patient comes first” approach of their employer, but clearly, the core of the Hippocratic Oath. This situation frequently leads to perceiving physician leaders in healthcare organization primarily—if not solely—as physicians, which further empowers the physician identity over that of the leader identity. It may be argued that physician leadership has an ultimately Hippocratic core via a collegial and communal spirit because the obligation to help resonates both with the Hippocratic Oath and the core concept of leadership (e.g. Ciulla, 1998 ). However, most definitions of leadership build on the idea of it being a process of intentional influence to organize, guide and facilitate actions and interpersonal relations in an organization or a group ( Yukl, 2013 ). While this is an apt description of physicians' leadership work, the Hippocratic core sets an important undertone to it.

The fifth dilemma of physician leadership relates to the inseparable ethical basis of the physician profession, reflected in the driving principle of “the patient comes first”. This orientation, forming the ethical foundation of the physician identity, means that the ethical expectations of the physician profession obligate physician leaders to continue patient work if needed. While time can be crucial to a patient, leadership/management work can be put aside for a moment, as it is an endless endeavour in organization, healthcare or otherwise. The dilemma here is how to best reconcile the ethical basis of the two identities.

## Conclusion

Even though most definitions of general leadership loosely fit the concept of physicians' leadership work, physician leadership appears to have two specific aspects, setting it apart from usual definitions, that may explain some of the challenges faced by physician leaders. First, the work of physician leaders does not only contain leadership and management work, but instead, physician leaders are intimately involved with the same work (patient work) as their subordinates, and they seem to habitually go beyond that by prioritizing patient work over leadership work. Second, there appears to be a widely spread undervaluation of the leadership aspect of what physician leaders do in the healthcare organization in comparison to patient work, which does not fit the definitions of leadership theories of general leadership (see also Quinn and Cola, 2020 ). These dilemmas are linked to the identity conflict physician leaders constantly experience in their work.

To offer viewpoints for future research on physician leadership and its practice, we first present practical implications based on our study. These implications, we hope, will be helpful in developing physician leaders to be more effective in their position to benefit the healthcare systems and their ultimate goal of positive patient outcomes. First, positive relationships are argued to support individuals in accepting a second identity as a leader ( Quinn and Cola, 2020 ). As physician leaders strongly identify with the profession of a physician, they would need firm enough footing in leadership skills, and professional knowledge related to leadership, to empower their leader identity and allow for a balance between the two identities.

In order to allow for a more holistic orientation towards leading healthcare organizations, both physician/specialist education and continuing education for practicing physician leaders should give equal weighting to the management and leadership aspect of the work. Therefore, healthcare organizations should reinforce physician leaders' professionalism by offering more practical leadership work training for physician leaders. This would equip physician leaders to more effectively close the gap between the competing worlds of medicine and management/leadership, as well as foster the development of healthcare systems (see Witman *et al.* , 2011 ) and develop their personal leader/manager identities.

This would necessitate physician leaders giving up some of their clinical duties to better concentrate on their leadership role. Accompanied by organization-wide leadership development, this would lead to a better command of leadership skills and strengthen the leader identity of physician leaders so that they would be able to better serve their organizations now and in the future ( Leatt and Porter, 2003 ). As the general leadership/management responsibilities of physicians increase, healthcare organizations could help physician leaders accept the leadership identity by allowing and organizing more peer group support for them, and by doing so, help create a stronger social group identity for physician leaders. This would further influence healthcare organizations, including the physicians being led, to respect physician leadership more and help leaders to be recognized as leaders and not “just” as physicians. The better physician leaders appraise themselves, the better the organization they work for accepts their leadership work (see Mintzberg, 1983a , pp. 4–5).

Furthermore, as Spehar *et al.* (2015) suggest, healthcare organizations need to not only focus on role and identity but also on need satisfaction when developing clinicians into leaders. Healthcare organizations should also support the growth of the clinicians' complex physician–leader identities, as work satisfaction supports productivity at work ( Zelenski *et al.* , 2008 ). Moreover, as Quinn and Perelli (2016) point out, role internalization, relational recognition and organizational endorsement are pivotal precursory elements for physician leaders' self-identity construction. This means that by recognizing and acknowledging the dual identity of a physician leader, healthcare organizations can succeed in their leadership performance for the benefit of not only the organization but the individual as well.

Since engaging physicians in the leadership of health systems affects staff and patient outcomes ( Onyura *et al.* , 2019 ) and improves patient care and organizational performance ( Falcone and Santiani, 2008 ; Goodall, 2011 ; Perry *et al.* , 2017 ; Spurgeon *et al.* , 2015 ; Tasi *et al.* , 2017 ), there is a need to solidify the meaning and value of physician leadership. This not only applies to physicians and physician leaders but to the whole healthcare organization. Therefore, in order to empower the professional identity of physician leaders as equal members of the professional community and not “just” as physicians, an attitude change is required for physicians to work as fully accepted formal leaders and not as “just” leaders or “just” physicians. This also applies to physicians as a profession. If physicians are willing to accept only physicians as their leaders, the medical discourse needs toning down and physician leaders need to be recognized as valuable members of the professional community, not judged as outcasts who have lost their passion for medicine (see also Styhre *et al.* , 2016 ).

Because the dual role of physician leaders as physicians and leaders seems to be irreversible, the resultant hybrid identity should be seen not as an unresolvable contradiction, but as complementary identities instead, offering the possibility for better physician leadership through resolving the dilemmas outlined in this study. Resolving these dilemmas will not be easy but is the key to achieving effective physician leadership and unlocking the improvement potential of health systems and their outcomes.

## Limitations and future research

The findings of our study should be considered with the following limitations: (1) participation was limited to physician leaders at one central hospital level hospital and (2) the sample size was small ( *n* = 23). In the future, it would be worthwhile to study challenges in physician leadership not only in university hospitals but also in the primary healthcare hospitals, taking into account the possible differences between various types of healthcare unit to gain more detailed understanding of the contextual issues (including national differences) and their effect on medical leadership.
